# Synergistic Ni Single Atoms/Nanoparticles on CeO_2_ for High‐Performance and Durable SOFC Hydrogen Electrodes

**DOI:** 10.1002/advs.76282

**Published:** 2026-06-30

**Authors:** Mengting Liu, Fengyang Yu, Qian Yang, Li Li, Yuesong Shen, Ling Huang

**Affiliations:** ^1^ State Key Laboratory of Chemistry and Utilization of Carbon Based Energy Resources College of Chemistry Xinjiang University Urumqi Xinjiang China; ^2^ School of Materials Science and Engineering Nanjing Tech University Nanjing China

**Keywords:** anode, hydrogen oxidation reaction, MOF engineering, single atoms/nanoparticles, solid oxide fuel cell

## Abstract

Degradation of the three‐phase boundaries in Ni‐based anodes at high operating temperature compromises hydrogen oxidation reaction catalytic activity and shortens the lifespan of solid oxide fuel cells. Herein, an oxide‐based anode decorated with Ni single atoms (SAs) and nanoparticles (NPs), i.e., e‐Ni/CeO_2_–5%, is synthesized by combining engineering of UiO‐66(Ce) metal organic frameworks (MOFs) and exsolution, where (i) Ni SAs donate electrons to generate oxygen vacancies and stabilize Ni NPs, (ii) NPs dissociate H_2_ to facilitate hydrogen spillover, and (iii) the abundant mesoporous channels in CeO_2_ inherited from the unique MOFs structure enables high‐efficient mass transfer. Synergy of the above merits finally delivers a greatly improved peak power density of 1.46 W cm^−2^ at 800°C among oxide‐based anodes, exhibits remarkable durability with negligible voltage degradation (< 0.005% h^−1^) over 500 h.

## Graphical abstract

Combination of UiO‐66(Ce) metal organic framework‐templated synthesis and in‐situ exsolution creates porous CeO_2_‐based anode decorated with Ni single atoms/nanoparticles, synergy of which leads to enhanced electronic/ionic transport, accelerated H_2_ oxidation, improved stability, and finally outstanding peak power density among oxide‐based SOFC anodes.

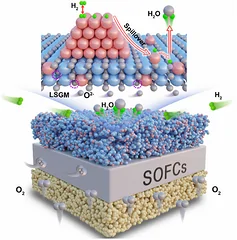

## 1 Introduction

Owing to the low/clean emissions, high efficiency, and wide fuel flexibility/availability, solid oxide fuel cells (SOFCs) represent a promising technology for directly converting chemical energy into electricity, helping to address climate change and energy shortages [1, 2]. Hydrogen is widely considered the ideal SOFC fuel due to its zero‐carbon footprint and high energy density. The overall performance of these systems is fundamentally governed by the hydrogen oxidation reaction (HOR) occurring at the anode. While Ni serves as the workhorse catalyst due to its high activity and cost‐effectiveness, it is the formation of three‐phase boundaries (TPBs) at the interface of the Ni‐based anode, electrolyte, and fuel gas that has proven highly critical for catalyzing HOR [3, 4, 5]. However, significant challenges persist because Ni particles are susceptible to sintering and agglomeration at high temperatures, which destroys the TPBs, degrades the output performance, shortens operational lifespan, and thus remains a core bottleneck for practical applications [6, 7]. Therefore, it is crucial to improve the structural durability of the catalyst while ensuring its catalytic activity.

Among various candidates, perovskites have become prevalent anode materials, particularly when combined with nanoparticles (NPs) in‐situ exsolved [4]. This composite structure, together with the electrolyte and fuel gas, forms a rich distribution of TPBs. However, perovskites exhibit relatively lower catalytic activity and electronic conductivity than those of Ni‐based analogues, leading to increased anode polarization, and consequently to low peak power density (PPD) [8, 9]. Even though high‐temperature operation (> 800°C) can partially offset this performance loss, it also causes accelerated migration and coarsening of the NPs and severe degradation of the TPB network, which drastically increases the polarization resistance (R_p_) [10, 11, 12, 13]. These challenges are compounded by high fabrication costs and poor mechanical properties, further complicating large‐scale production and stack assembly.

**FIGURE 1:**
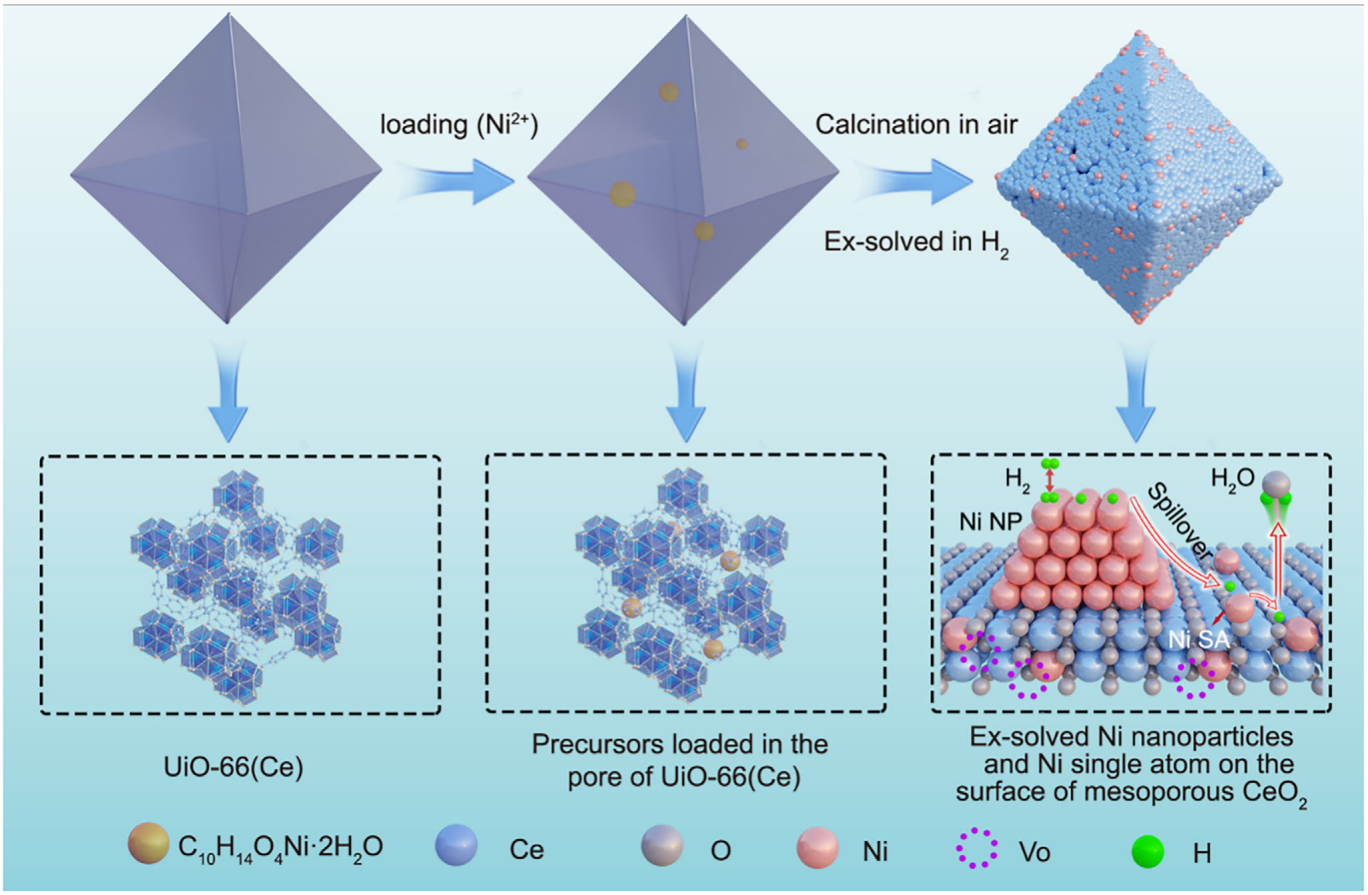
Schematics of synthesis procedures to obtain porous Ni/CeO_2_ catalysts functionalized with Ni SAs/NPs from UiO‐66(Ce).

To tackle the above challenges, a CeO_2_‐based oxide anode, engineered from a UiO‐66(Ce) metal‐organic framework (MOF) precursor is developed. As illustrated in Figure 1, Ni^2+^ ions are first anchored within the MOF pores by coordinating with carboxylic groups in the BDC (C_8_H_6_O_4_) linker, and/or at the open metal sites on the framework by immersing the pre‐synthesized MOF in Ni^2+^ solution [14, 15]. Subsequent calcination and reduction in an H_2_ atmosphere yields a CeO_2_‐based anode co‐hosting Ni single atoms (SAs) and NPs. In this architecture, the Ni SAs drive the formation of oxygen vacancies (*V_O_* s) through charge compensation, whereas the Ni NPs are firmly anchored on the CeO_2_ surface via strong metal‐support interactions [4, 16, 17, 18, 19, 20], while Ni NPs concurrently dissociate H_2_ to facilitate hydrogen spillover [21, 22]. This unique architecture offers multiple advantages: (i) reduced overall R_p_ because the electrochemically active region expands from narrow TPB lines to the entire CeO_2_ surface [23], (ii) mitigated anode overpotential where the improved mixed ionic‐electronic conductivity (MIEC) of the CeO_2_ host fosters abundant active sites and accelerated reaction kinetics [24, 25], (iii) the hierarchical architecture integrating macroscopic gas‐diffusion channels with MOF‐inherited mesoporous surfaces, confines the Ni NPs effectively and accelerates local surface reaction kinetics, (iv) the synergy of Ni SAs and NPs with the porous CeO_2_ host leads to a PPD of 1.46 W cm^−2^ at 800°C in an La_0.9_Sr_0.1_Ga_0.8_Mg_0.2_O_3_ (LSGM) electrolyte‐supported single cell, with negligible degradation (< 0.005% h^−1^) during a 500‐h continuous operation.

## 2 Results and Discussion

**FIGURE 2:**
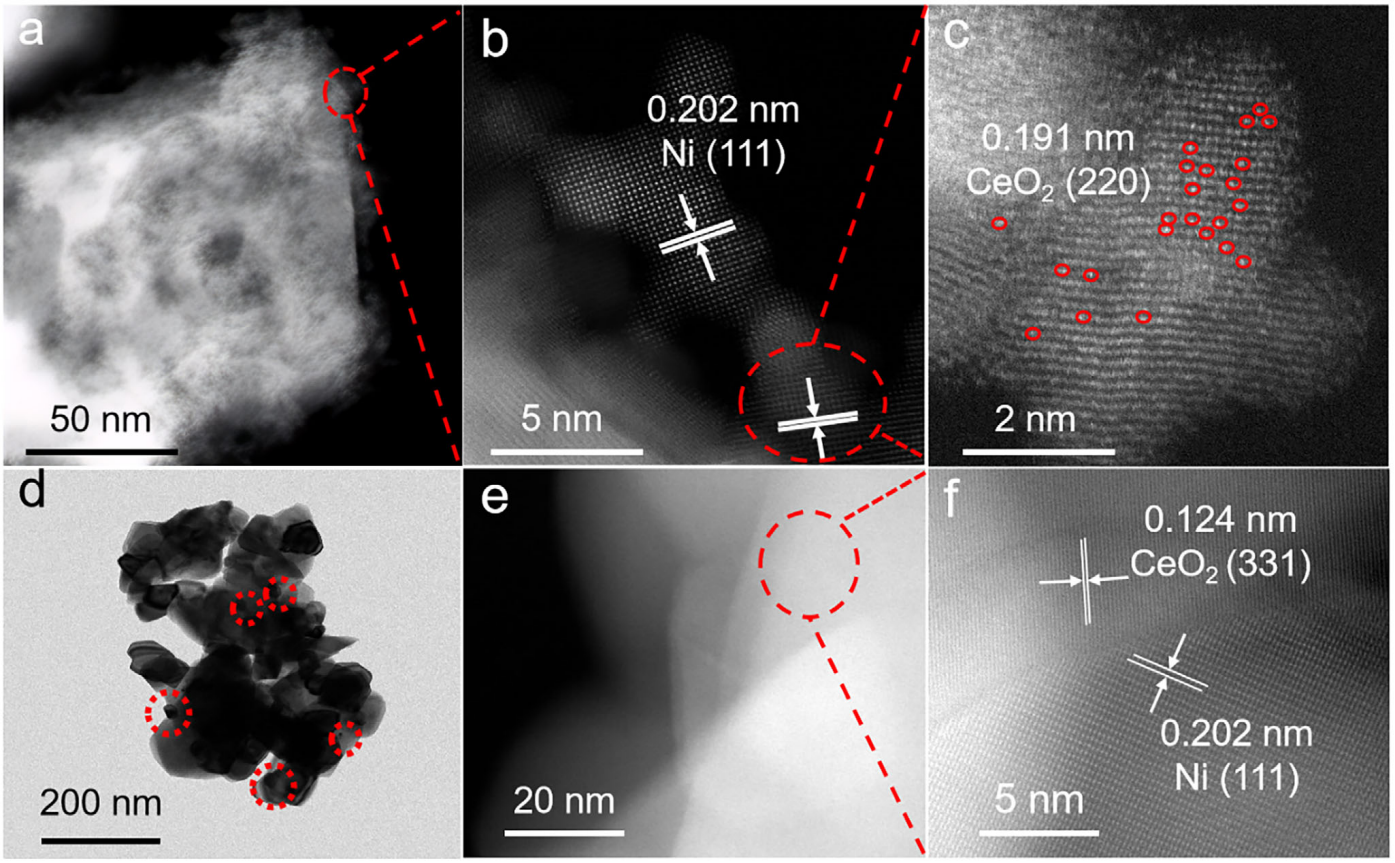
(a) HAADF‐STEM image showing (b) characteristic lattice of Ni NPs and (c) Ni SAs indicated by red circles in e‐Ni/CeO_2_–5%. (d‐f) HAADF STEM images of i‐Ni/CeO_2_–5% powder following the identical treatment.

Control experiments indicate that the sample with optimized catalytic performance was obtained by impregnating the porous UiO‐66(Ce) (Figure S1) framework with a 5 mol% Ni^2+^ solution, i.e., e‐Ni/CeO_2_–5%. After pyrolysis at 800°C under an air atmosphere, the MOF precursor transformed into a porous CeO_2_ structure, with Ni penetrating into the bulk and subsurface lattices to form a Ce–Ni–O solid solution (Figure S2a–d) [14]. High‐angle annular dark‐field scanning transmission electron microscopy (HAADF‐STEM) analyses indicate that a subsequent reduction at 800°C (5% H_2_/Ar) triggered the exsolution of Ni species, with a portion of Ni atoms diffusing to the surface to form uniform Ni NPs with an average size of 2–6 nm (Figure 2a,b). Concurrently, the remaining Ni atoms were trapped at surface defects, predominantly *V_O_*, as stabilized SAs (Figure 2c). This configuration establishes a strong metal‐support interaction, which is crucial for stabilizing the SAs and preventing NP sintering [15]. Furthermore, the reduction of Ce^4+^ to Ce^3+^ during the exsolution process is accompanied by the generation of abundant *Vo* to maintain charge neutrality [26]. The resultant increase in Ce^3+^ concentration significantly enhances the n‐type electronic conductivity of the CeO_2_ matrix, rendering it a highly effective MIEC. This characteristic broadens the electrochemically active zone beyond the conventional TPB, a critical advancement in SOFC nanoscience [27]. Capitalizing on this enhanced conductivity, the exsolution strategy bypasses the typical TPB construction limits associated with low catalyst loadings [4]. In this designed architecture, the continuous CeO_2_ matrix serves as the primary ionic conductor, while the exsolution process drives the Ni cations from the bulk lattice to the surface, leading to a significant surface enrichment effect. These in‐situ formed and firmly anchored Ni NPs act as highly efficient electronic conductors and catalytic sites. By uniformly decorating the surface of the CeO_2_ scaffold, they seamlessly integrate with the ionic conductor and the gas phase, constructing an extensively distributed and intimate TPB network. In stark contrast, the control sample obtained by immersing the porous UiO‐66(Ce) framework into 5 mol% Ni^2+^ solution, i.e., i‐Ni/CeO_2_–5%, exhibits only large, aggregated Ni NPs (>20 nm), with no observation of SAs (Figure 2d–f). Energy‐dispersive X‐ray spectroscopy (EDS) mapping reveals a highly homogeneous Ni distribution in e‐Ni/CeO_2_–5%, devoid of the large aggregates seen in its impregnated counterpart (Figure 2d and Figure S2e–l, marked by red circles). This excellent dispersion is attributed to the MOF‐inherited mesoporous scaffold, where the close match between the NP size (2–6 nm) and the mesopore diameter ensures the NPs are either embedded within or anchored to the channel walls [28]. Consequently, the X‐ray diffraction (XRD) peaks corresponding to metallic Ni are substantially weaker in e‐Ni/CeO_2_–5% (Figure S3a).

**FIGURE 3:**
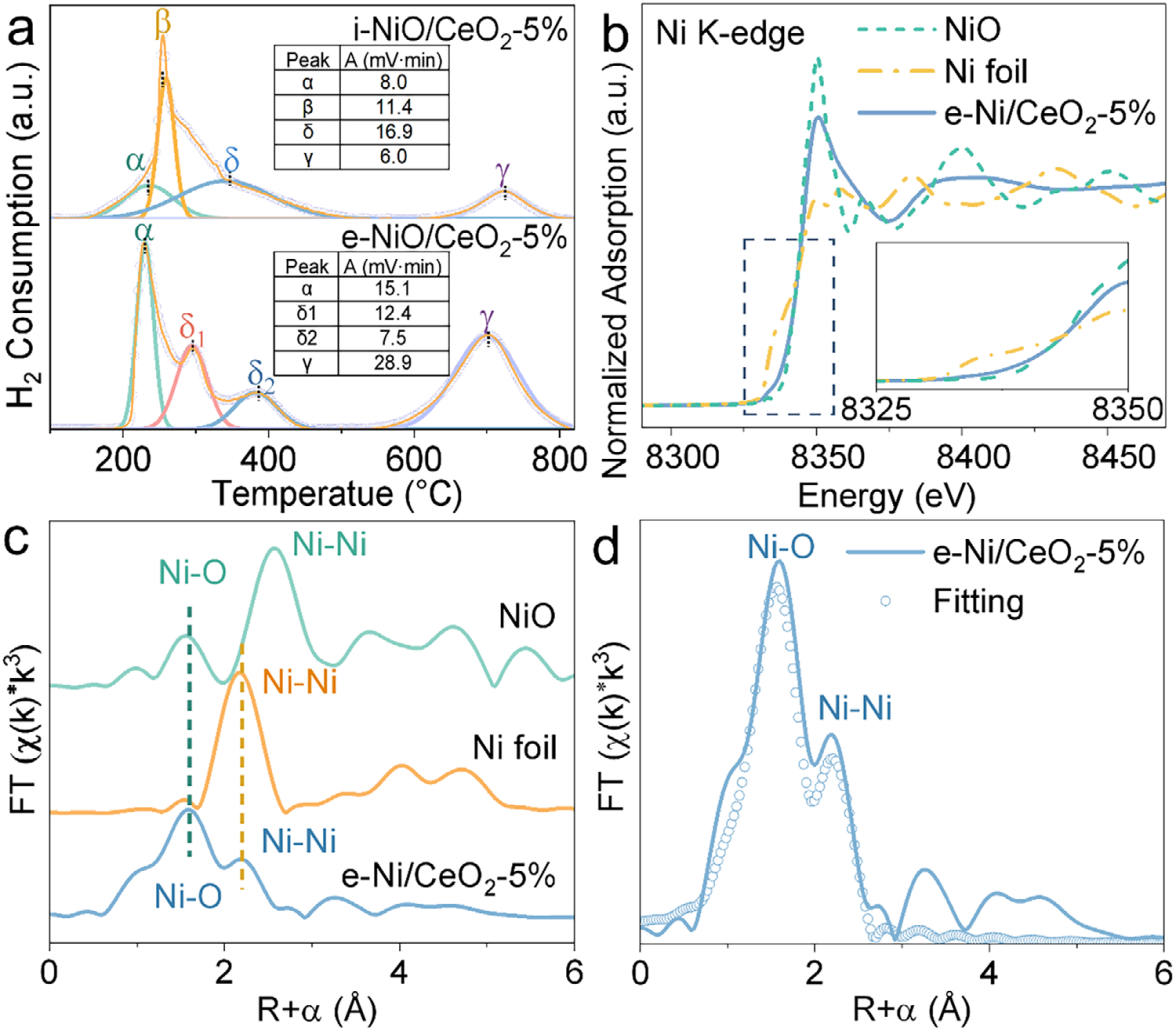
(a) H_2_‐TPR profiles of the Ni/CeO_2_ catalysts. (b) Ni K‐edge XANES spectra and (c) Ni K‐edge EXAFS spectra. (d) Experimental and fitting Ni K‐edge FT‐EXAFS spectra of e‐Ni/CeO_2_–5%.

The hydrogen temperature‐programmed reduction (H_2_‐TPR) profiles in Figure 3a exhibits four distinct reduction peaks, providing semi‐qualitative insights into the structural evolution and catalytic roles of Ni SAs/NPs in e‐NiO/CeO_2_–5% and i‐NiO/CeO_2_–5%. The sharp reduction α peak at ∼230°C can be ascribed to the reduction of adsorbed active oxygen species on the surface of CeO_2_ [29]. Combined with the XRD results (Figure S3b), e‐NiO/CeO_2_–5% shows no aggregated NiO diffraction peaks, which indicates that Ni^2+^ cations are doped into the CeO_2_ lattice, and resultantly, a large number of *V_O_* s are generated to maintain the charge neutrality. Meanwhile, the *V_O_* s generated through the formation of the Ni–O–Ce solid solution can adsorb additional oxygen species, thereby enhancing the α peak at 230°C for e‐NiO/CeO_2_–5% (the α relative peak area is 15.1 mV·min). This observation supports the active role of Ni^2+^ in promoting *V_O_* s formation in the catalyst. In contrast, the hydrogen consumption corresponding to the α peak (associated with adsorbed oxygen on vacancies) on e‐NiO/CeO_2_–5% (relative peak area 8.0 mV·min) is negligible. For e‐NiO/CeO_2_–5%, two resolved peaks are observed at δ_1_ (295°C) and δ_2_ (382°C), corresponding to consecutive reduction steps: the δ_1_ peak is attributed to the reduction of Ni‐SAs formed by exsolution from the solid solution, while the δ_2_ peak is associated with the formation of well‐dispersed Ni NPs [30, 31]. Compared with to the impregnation‐prepared i‐NiO/CeO_2_–5%, the shift of the reduction peaks of e‐NiO/CeO_2_–5% to higher temperatures indicates significantly enhanced metal‐support interactions [30, 31], which effectively suppresses Ni NPs sintering and improves the structural stability of the material. In contrast, the β peak at 260°C and the δ peak at 340°C for i‐NiO/CeO_2_–5% suggest that the conventional impregnation method leads to weaker metal‐support interactions and agglomeration of bulk‐like NiO species on the surface, making them easier to be reduced. The γ peak in the high‐temperature region (≥700°C) corresponds to the reduction of bulk lattice oxygen in CeO_2_ [31]. Notably, the reduction temperature of Ce─O bonds in e‐NiO/CeO_2_–5% (700°C) shifts to a lower temperature compared with that of i‐NiO/CeO_2_–5% (723°C), indicating that Ni doping weakens the Ce─O bond. Furthermore, the significantly larger relative peak area of the γ peak for e‐NiO/CeO_2_–5% (28.9 mV·min vs. 6 mV·min for i‐NiO/CeO_2_–5%) confirms that small, well‐dispersed Ni NPs, formed via the exsolution process, enhance hydrogen spillover at the metal‐support interface [32, 33]. In summary, the synergistic interaction (such as electron transfer) between exsolved Ni NPs and SAs in e‐NiO/CeO_2_–5% significantly enhances the reducibility activity of CeO_2_ and the generation of *V_O_* s and Ce^3+^ species, thereby enhancing their ionic and electronic conductivity. Meanwhile, this strong metal‐support interaction (mediated by Ni─O─Ce bonds) stabilizes Ni NPs, thereby avoiding the common Ni NP agglomeration in Ni‐based SOFCs to a certain extent and potentially improving the long‐term stability of the cell.

The X‐ray absorption near edge structure (XANES) spectra (Figure 3b) provide insights into the oxidation states of Ni SAs/NPs in the catalyst. The absorption edge energy of the sample lies between that of Ni foil (Ni^0^) and NiO (Ni^2+^), indicating an average Ni oxidation state between 0 and +2. This intermediate valence state suggests a potential electronic interaction between Ni SAs and NPs, which could modulate their respective electronic structures [34]. This observation is further supported by extended X‐ray absorption fine structure (EXAFS) spectroscopy (Figure 3c,d), where characteristic scattering paths corresponding to Ni–O (∼1.6 Å) and Ni–Ni (∼2.1 Å) in the R‐space (without phase correction) can be assigned to Ni–O and Ni–Ni coordination, corresponding to isolated SAs and NPs, respectively [21, 35]. These findings are supported by quantitative EXAFS fitting and wavelet‐transform analysis (Figures S4 and S5).

**FIGURE 4:**
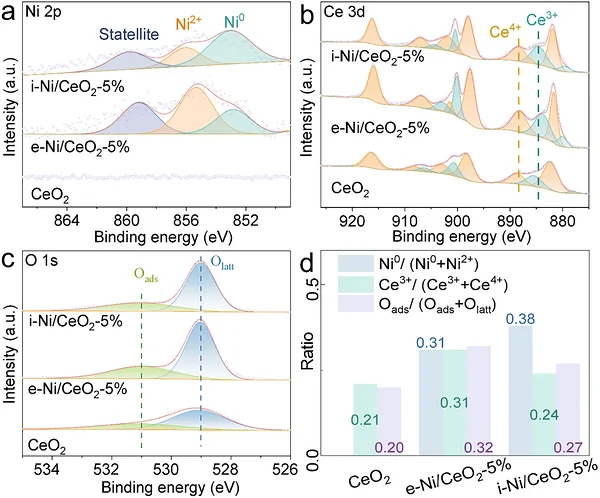
XPS spectra of (a) Ni 2p, (b) Ce 3d and (c) O 1s for the reduced Ni/CeO_2_ catalysts. (d) Ratios derived from XPS analysis.

X‐ray photoelectron spectroscopy (XPS) analysis elucidates the oxidation states of Ni SAs/NPs and the associated *V_O_* concentration in the catalysts. In e‐Ni/CeO_2_–5%, the reduction process generates both metallic Ni^0^ and isolated Ni^2+^, accompanied by partial reduction of the CeO_2_ surface and *V_O_* formation. The Ni 2p spectra (Figure 4a and Figure S6) exhibit a clear Ni^0^ signal, confirming metallic Ni on the surface. Notably, the relative Ni^0^ content in e‐Ni/CeO_2_–5% is lower than that in the i‐Ni/CeO_2_–5%, suggesting incomplete reduction of Ni species to the metallic state. This can be attributed to the formation of a Ni–O–Ce solid solution and strong metal‐support interactions at the Ni‐CeO_2_ interface, which stabilize Ni in a higher oxidation state [21, 22, 23, 24, 25, 36]. The deconvoluted Ce 3d spectra in Figure 4b can be assigned to the 3d^10^4f^1^ and 3d^10^4f^0^ final states of Ce^3+^ and Ce^4+^ species, respectively [37]. Since Ce^3+^ formation is coupled with *V_O_* generation, the Ce^3+^/(Ce^3+^+Ce^4+^) ratio is a reliable indicator of the overall *V_O_* concentration [38]. The O 1s spectrum (Figure 4c) can be deconvoluted into two main components: lattice oxygen (O_latt_, O^2−^) at 529.01 eV and surface‐adsorbed oxygen species (O_ads_) at 531.30 eV [39]. The concentration of surface *V_O_* can be reflected by calculating the ratio of O_ads_/(O_latt_+O_ads_) [40]. The proportions of both Ce^3+^ and O_ads_ are significantly higher in e‐Ni/CeO_2_–5% than those in i‐Ni/CeO_2_–5% and pure CeO_2_ (Figure 4d), consistently indicating a substantially increased *V_O_* concentration. The strong electronic interactions between highly dispersed isolated Ni sites and the CeO_2_ support induce the formation of asymmetric Ce‐O‐Ni structures [41, 42], which weaken Ce─O bonds and facilitate *V_O_* generation, thereby activate adjacent Ce sites by increasing the Ce^3+^/Ce^4+^ ratio. Moreover, these abundant *V_O_* sites drive electron transfer from CeO_2_ to Ni, yielding an electron‐rich Ni surface [25, 43]. This charge transfer, in synergy with hydrogen spillover from the well‐dispersed Ni NPs, further facilitates the reduction of CeO_2_ [44, 45]. Consequently, such structural optimization of the anode is anticipated to mitigate polarization losses, providing a strong rationale for evaluating the cell's electrochemical performance and long‐term stability.

**FIGURE 5:**
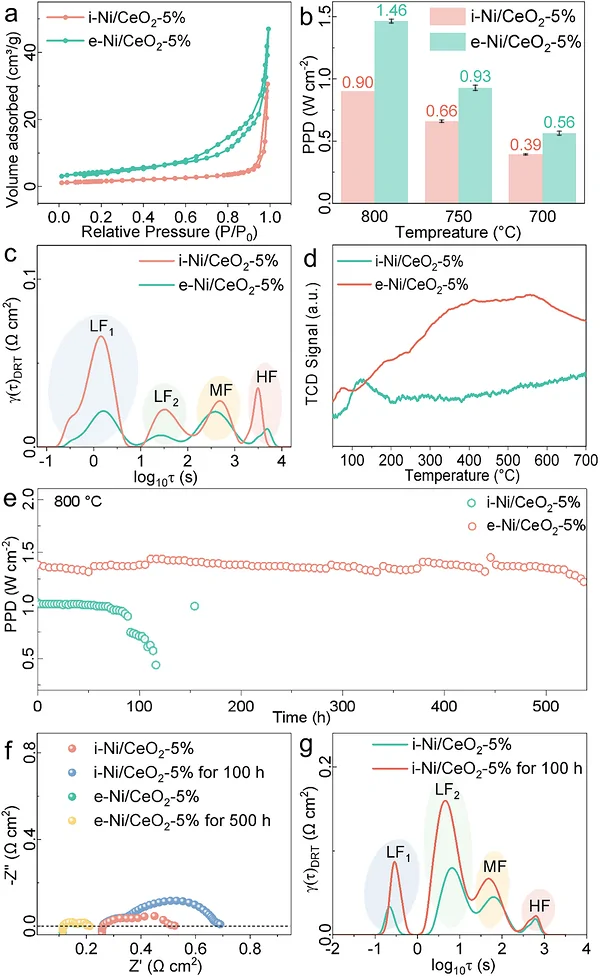
(a) N_2_ adsorption and desorption isotherms of e‐Ni/CeO_2_–5% and i‐Ni/CeO_2_–5%. (b) PPD of single cells with the Ni/CeO_2_ anodes within the temperature range of 700°C –800°C. (c) DRT analysis of the single cell with Ni/CeO_2_ anode operated in wet H_2_ (3 vol.% H_2_O) at 800°C. (d) H_2_‐TPD profiles of the Ni/CeO_2_ catalysts. (e) Long‐term stability tests of a single cell with e‐Ni/CeO_2_–5% and i‐Ni/CeO_2_–5% as anodes in wet H_2_ (3 vol.% H_2_O) at 800°C. (f) EIS spectra of the Ni/CeO_2_ catalysts before and after the long‐term stability tests. (g) DRT analyses of the cells with the i‐Ni/CeO_2_–5% anode before and after the long‐term stability tests.

Electrochemical impedance spectroscopy (EIS) in wet H_2_ (3 vol.% H_2_O) was collected on Ni/CeO_2_|LDC|LSGM|LDC|Ni/CeO_2_ symmetrical cells, at 800°C, 750°C, and 700°C, respectively, where LDC is used as a barrier layer and LSGM as an electrolyte (Figure S7). Compared with the R_p_ of 0.149 Ω cm^2^ in i‐Ni/CeO_2_–5% at 800°C, e‐Ni/CeO_2_–5% shows a remarkably lower R_p_ of only 0.098 Ω cm^2^ (Figure S8 and Table S1). This enhanced performance stems from a synergistic catalytic effect involving Ni SAs, Ni NPs [14, 29, 35, 41], as well as the abundant mesoporous channels in CeO_2_ framework (Figure 5a), which improves the accessibility of active sites and thereby accelerates the HOR kinetics [45].

To evaluate its performance in a practical SOFC, an electrolyte‐supported single cell of Ni/CeO_2_|LDC|LSGM|LDC|LSCF (La_0.6_Sr_0.4_Co_0.2_Fe_0.8_O_3‐δ_) was fabricated (Figure S9) with corresponding layer thicknesses of 30 µm for the anode, 15 µm for the LDC barrier, 300 µm for the LSGM electrolyte, and 20 µm for the LSCF cathode, respectively. Remarkable PPD of 1.46, 0.93 and 0.56 W cm^−2^ in wet H_2_ (3 vol.% H_2_O) at 800°C, 750°C, and 700°C, respectively, were obtained using e‐Ni/CeO_2_–5% as anode (Figure 5b and Figure S10a,b,c and Table S2). Notably, the PPD of 1.46 W cm^−2^ at 800°C remains the highest value among oxide‐based anodes (Table S4). EIS analysis at open‐circuit voltage reveals a low total resistance (R_total_ = R_o_ + R_p_), consisting of an ohmic resistance (R_o_) of 0.113 Ω cm^2^ and a R_p_ of 0.099 Ω cm^2^ (Figure S10d,e,f and Table S3). Compared with that of the i‐Ni/CeO_2_–5%, the observed reduction in R_o_ (0.177 Ω cm^2^) can be ascribed to the improved interfacial contact facilitated by the fine exsolved Ni NPs and Ni SAs covering the CeO_2_ surface, together with Ni^2+^ doping in the lattice. Furthermore, compared with the agglomeration in the i‐Ni/CeO_2_–5%, the fine Ni NPs uniformly spreading over the surface of e‐Ni/CeO_2_–5% supply more electronic conduction pathways within the anode. Although strictly isolating the individual contribution of Ni SAs is challenging due to concurrent microstructural changes (e.g., porosity, *V_O_* content), the tailored Ni SA/NP‐CeO_2_ architecture clearly goes beyond classical charge and mass transport. This highly coupled ionic‐electronic network, as described by the State‐Resonant Energy Transmission Law (SRETL) [46], enables resonance‐mediated energy transmission alongside classical migration. This synergy leads to significantly reduced R_o_ and R_p_, and ultimately, superior PPD.

The hydrogen spillover mechanism for HOR at the TPBs of Ni/YSZ anode is well‐established [6]. Building on this framework, we investigated the enhanced kinetics of HOR over Ni/CeO_2_ anodes in SOFCs. The distribution of relaxation times (DRT) analysis of the EIS data suggests that the superior performance of e‐Ni/CeO_2_–5% is associated with a holistic, multi‐stage enhancement of the HOR process, which involves four key steps (Figure 5c). HF (High‐frequency, 10^3^–10^4^ Hz) corresponds to the charge transfer process at the TPBs [6], which primarily involves electron transfer and oxygen‐ion conduction through the CeO_2_ matrix. The significantly smaller peak area for e‐Ni/CeO_2_–5% than for i‐Ni/CeO_2_–5% indicates superior electrochemical kinetics, resulting from a substantially lower charge transfer resistance [36, 45]. MF (Medium‐frequency, 10^2^–10^3^ Hz) represents electrochemical dissociative adsorption of H_2_, which is preferentially facilitated on metal surfaces with high d‐electron state density, such as Ni NPs [47]. Owing to its highly dispersed Ni NPs, e‐Ni/CeO_2_–5% exhibits a markedly lower impedance in this frequency range than its impregnated counterpart. LF_2_ (Low‐frequency, 10^1^–10^2^ Hz) indicates surface diffusion of adsorbed hydrogen species (H^*^) on CeO_2_, facilitated by hydrogen spillover [3, 47]. This assignment, along with the identification of diffusion as the rate‐limiting step, was rigorously verified by varying the H_2_ partial pressure and temperature; the equivalent circuit fitting (Figure S11a) and its alignment with DRT results (Figure S11b) confirm the dominance of diffusion‐related resistance. Consistent with this, the hydrogen adsorption capacity of e‐Ni/CeO_2_–5% is significantly greater than that of i‐Ni/CeO_2_–5% (Figure 5d). This enhancement is attributed to the synergy between Ni SAs (for H* anchoring) and Ni NPs (for H_2_ dissociation), which collectively enhance the overall hydrogen adsorption and diffusion via a spillover mechanism (Figure S12). LF_1_ (10^−1^–10^1^ Hz) means gas‐phase diffusion within the anode pores, which remains a unique advantage of the MOF‐derived porous nanostructure (Figure 5a) [47].

Hydrogen temperature‐programmed desorption (H_2_‐TPD) analysis provides critical evidence for decoupling the distinct roles of Ni NPs, Ni SAs, and their composite effect (Figure 5d). As a baseline, the i‐Ni/CeO_2_–5% catalyst exhibits only a single desorption peak at  130°C, which is attributed to hydrogen directly adsorbed and desorbed on the surface of conventional Ni NPs [48]. In stark contrast, e‐Ni/CeO_2_–5% displays a broad and intense desorption peak at approximately 450°C. This high‐temperature feature is a definitive evidence of the significant hydrogen spillover process driven by the composite structure. In this synergistic architecture, the highly dispersed Ni NPs act as the primary sites for H_2_ dissociation. Crucially, the atomically dispersed Ni SAs play a distinct role: they strongly modulate the electronic structure of the surrounding CeO_2_ matrix (as confirmed by XPS), creating abundant *V_O_*. Consequently, the hydrogen dissociated on the NPs rapidly spills over onto the SA‐modified CeO_2_ support, forming strongly adsorbed hydrogen species that require elevated temperatures for desorption (Figure S12). The substantial magnitude of this 450°C peak, compared to the mere 130°C peak in i‐Ni/CeO_2_–5%, underscores how the strong metal‐support interaction, specifically initiated by the SAs, radically enhances the redox properties and hydrogen handling capability far beyond what isolated NPs can achieve.

While exceptional catalytic activity is a prerequisite, long‐term stability under practical operating conditions is equally critical. A primary challenge is the sintering of Ni NPs at high temperature, driven by their high surface energy, which leads to the degradation of catalytic activity and operational durability. A comparative stability test conducted at 800°C in wet H_2_ shows that i‐Ni/CeO_2_–5% anode succumbed to rapid performance degradation within just 70 h of operation (Figure 5e), whereas e‐Ni/CeO_2_–5% anode demonstrates over ∼500 h stability, with a negligible PPD degradation rate of <0.005% h^−1^. Analysis of the EIS evolution before and after the stability test (Figure 5f,g) provides further insight. The impedance associated with hydrogen dissociation and surface diffusion increased significantly for i‐Ni/CeO_2_–5% after testing. This increase is consistent with severe Ni NP agglomeration (indicated by white circles in Figure S13a–d) and the consequent loss of active TPBs. Conversely, the superior durability of e‐Ni/CeO_2_–5% originates from the strong interaction between the active Ni SAs and NPs and the porous CeO_2_ support, which is stabilized by the Ce‐O‐Ni structure and effectively inhibits high‐temperature Ni NP sintering (Figure S13e–h) [16, 17, 18, 19, 20, 41, 42].

## 3 Conclusions

A novel oxide‐based anode, e‐Ni/CeO_2_–5%, has been developed through MOF engineering. The porous scaffold provides abundant and continuous electron/ion transport paths, high surface area allows more SAs and NPs with exceptional catalytic activity, while strong metal‐support interaction ensures long‐term operational stability, and synergy of which finally leads to optimized charge transfer (R_P_ = 0.098 Ω cm^2^), accelerated hydrogen spillover, improved HOR kinetics, excellent stability, and outstanding PPD of 1.46 W cm^−2^ at 800°C. This work provides a novel angle to fabricating oxide‐based high‐performance anodes, directly addressing the key challenges in SOFC commercialization.

## Author Contributions

**Yuesong Shen**: conceptualization, validation. **Li Li**: investigation, validation. **Qian Yang**: investigation, formal analysis, visualization. **Ling Huang**: conceptualization, supervision, writing – review and editing. **Mengting Liu**: data curation, validation, investigation, methodology, writing – original draft. **Fengyang Yu**: conceptualization, formal analysis, writing – original draft.

## Conflicts of Interest

The authors declare no conflicts of interest.

## Supporting information




**Supporting file**: advs76282‐sup‐0001‐SuppMat.docx

## Data Availability

The data that supports the findings of this study are available in the supplementary material of this article.

## References

[1] E. Wachsman and K. Lee , “Lowering the Temperature of Solid Oxide Fuel Cells,” Science 334 (2011): 935–939, 10.1126/science.1204090. 22096189

[2] X. Liu , D. Xie , J. T. S. Irvine , J. Ni , and C. Ni , “An FeNbO_4_‐Based Oxide Anode for a Solid Oxide Fuel Cell (SOFC),” Electrochimica Acta 225 (2020): 135692, 10.1016/j.electacta.2020.135692.

[3] I. Jang , J. S. A. Carneiro , J. O. Crawford , et al., “Electrocatalysis in Solid Oxide Fuel Cells and Electrolyzers,” Chemical Reviews 124 (2024): 8233–8306, 10.1021/acs.chemrev.4c00008. 38885684

[4] Y. Liu , M. Juckel , N. H. Menzler , and A. Weber , “Ni/GDC Fuel Electrode for Low‐Temperature SOFC and Its Aging Behavior under Accelerated Stress,” Journal of the Electrochemical Society 171 (2024): 054514, 10.1149/1945-7111/ad4917.

[5] P. Sawant , S. Varma , M. R. Gonal , B. N. Wani , D. Prakash , and S. R. Bharadwaj , “Effect of Ni Concentration on Phase Stability, Microstructure and Electrical Properties of BaCe_0.8_Y_0.2_O_3‐δ_‐Ni Cermet SOFC Anode and Its Application in Proton Conducting ITSOFC,” Electrochimica Acta 120 (2014): 80–85, 10.1016/j.electacta.2013.12.061.

[6] Q. Fu , C. Tian , L. Hun , et al., “Ni Agglomeration and Performance Degradation of Solid Oxide Fuel Cell: a Model‐Based Quantitative Study and Microstructure Optimization,” Energy 289 (2024): 129997, 10.1016/j.energy.2023.129997.

[7] O. Rahumi , Y. Yuferov , L. Meshi , N. Maman , and K. Borodianskiy , “Ni‐Doping Strategy for Perovskite Anodes towards High‐Performance Ammonia‐Fueled SOFCs,” Journal of Power Sources 631 (2025): 236320, 10.1016/j.jpowsour.2025.236320.

[8] C. Duan , R. J. Kee , H. Zhu , et al., “Highly Durable, Coking and Sulfur Tolerant, Fuel‐Flexible Protonic Ceramic Fuel Cells,” Nature 557 (2018): 217–222, 10.1038/s41586-018-0082-6. 29743690

[9] Y. Wan , Y. Xing , Z. Xu , S. Xue , S. Zhang , and C. Xia , “A‐Site Bismuth Doping, A New Strategy to Improve the Electrocatalytic Performances of Lanthanum Chromate Anodes for Solid Oxide Fuel Cells,” Applied Catalysis B: Environmental 269 (2020): 118809, 10.1016/j.apcatb.2020.118809.

[10] D. E. Fowler , A. C. Messner , E. C. Miller , B. W. Slone , S. A. Barnett , and K. R. Poeppelmeier , “Decreasing the Polarization Resistance of (La,Sr)CrO_3−δ_ Solid Oxide Fuel Cell Anodes by Combined Fe and Ru Substitution,” Chemistry of Materials 27 (2015): 3683–3693, 10.1021/acs.chemmater.5b00622.

[11] J. Karczewski , B. Riegel , M. Gazda , P. Jasinski , and B. Kusz , “Electrical and Structural Properties of Nb‐doped SrTiO_3_ Ceramics,” Journal of Electroceramics 24 (2010): 326–330, 10.1007/s10832-009-9578-7.

[12] Y. Zhang , H. Zhao , Z. Du , K. Swierczek , and Y. Li , “High‐Performance SmBaMn_2_O_5+δ_ Electrode for Symmetrical Solid Oxide Fuel Cell,” Chemistry of Materials 31 (2019): 3784–3793, 10.1021/acs.chemmater.9b01012.

[13] Z. Cao , Y. Zhang , J. Miao , et al., “Titanium‐Substituted Lanthanum Strontium Ferrite as a Novel Electrode Material for Symmetrical Solid Oxide Fuel Cell,” International Journal of Hydrogen Energy 40 (2015): 16572–16577, 10.1016/j.ijhydene.2015.10.010.

[14] T. Pu , J. Chen , W. Tu , et al., “Dependency of CO_2_ Methanation on the Strong Metal‐Support Interaction for Supported Ni/CeO_2_ Catalysts,” Journal of Catalysis 413 (2022): 821–828, 10.1016/j.jcat.2022.07.038.

[15] Z. Q. Rao , K. W. Wang , Y. H. Cao , et al., “Light‐Reinforced Key Intermediate for Anticoking To Boost Highly Durable Methane Dry Reforming over Single Atom Ni Active Sites on CeO_2_ ,” Journal of the American Chemical Society 145 (2023): 24625–24635. 10.1021/jacs.3c07077 37792912

[16] J.‐H. Myung , D. Neagu , D. N. Miller , and J. T. S. Irvine , “Switching on Electrocatalytic Activity in Solid Oxide Cells,” Nature 537 (2016): 528–531, 10.1038/nature19090. 27548878

[17] X. Li , X. I. Pereira‐Hernández , Y. Chen , et al., “Functional CeO_x_ Nanoglues for Robust Atomically Dispersed Catalysts,” Nature 611 (2022): 284–288, 10.1038/s41586-022-05251-6. 36289341

[18] Z. H. Pei , H. B. Zhang , Z. P. Wu , et al., “Atomically Dispersed Ni Activates Adjacent Ce Sites for Enhanced Electrocatalytic Oxygen Evolution Activity,” Science Advance 9 (2023): adh1320. 10.1126/sciadv.adh1320 PMC10306285 37379398

[19] B. Tang , X.‐L. Zhang , Q. Ji , et al., “Investigation of the Relationship between Metal Loading and Acidic Oxygen Evolution Reaction Activity in Single‐Atom Catalysts,” ACS Catalysis 14 (2024): 3788–3797, 10.1021/acscatal.3c06263.

[20] X. Li , X. Zhang , Z. Du , et al., “Electronic Enrichment on Ni Atoms at Ni‐CeO_2_ Interfaces: Unraveling the Catalytic Role in CO Methanation and Its Volcano‐Type Relation With the CeO_2_ Content,” Chinese Journal of Catalysis 74 (2025): 177–190, 10.1016/S1872-2067(25)64688-2.

[21] M. Xu , M. Peng , H. Tang , W. Zhou , B. Qiao , and D. Ma , “Renaissance of Strong Metal–Support Interactions,” Journal of the American Chemical Society 146 (2024): 2290–2307, 10.1021/jacs.3c09102. 38236140

[22] Y. He , Y. Li , J. Zhang , et al., “Low‐Temperature Strategy toward Ni‐NC@Ni Core‐Shell Nanostructure With Single‐Ni Sites for Efficient CO_2_ Electroreduction,” Nano Energy 77 (2020): 105010, 10.1016/j.nanoen.2020.105010.

[23] Q.‐Y. Guo , Z. Wang , X. Feng , Y. Fan , and W. Lin , “Generation and Stabilization of a Dinickel Catalyst in a Metal‐Organic Framework for Selective Hydrogenation Reactions,” Angewandte Chemie International Edition 62 (2023): 202306905, 10.1002/anie.202306905. 37418318

[24] X. D. Nguyen , S. W. Lee , S. J. Kim , et al., “Boosting Electrochemical Performance via Extra‐Role of La‐Doped CeO_2‐δ_ Interlayer for “Oxygen Provider” at High‐Current SOFC Operation,” Advanced Science 11 (2024): 2402348, 10.1002/advs.202402348. PMC11633512 39331567

[25] C. Zhou , K. Wu , H. Huang , et al., “Spatial Confinement of Electron‐Rich Ni Nanoparticles for Efficient Ammonia Decomposition to Hydrogen Production,” ACS Catalysis 11 (2021): 10345–10350, 10.1021/acscatal.1c02420.

[26] Y. Chen , C. Deng , W. Luo , et al., “Concurrent Surface and Bulk Modification of Ceria‐based Cathodic Catalyst for CO_2_ Reduction in Solid Oxide Electrolysis Cell under Strong Polarization Conditions,” Journal of Advanced Ceramics 15 (2026): 9221219, 10.26599/JAC.2025.9221219.

[27] L. Fan and B. Zhu , “Nanomaterials and Technologies for Low Temperature Solid Oxide Fuel Cells: Recent Advances, Challenges and Opportunities,” Nano Energy 45 (2018): 148–176, 10.1016/j.nanoen.2017.12.044.

[28] K. Wan , J. Luo , C. Zhou , et al., “Hierarchical Porous Ni_3_S_4_ With Enriched High‐Valence Ni Sites as a Robust Electrocatalyst for Efficient Oxygen Evolution Reaction,” Advanced Functional Materials 29 (2019): 1900315, 10.1002/adfm.201900315.

[29] A. Alarcón , J. Guilera , G. Jordi , et al., “Higher Tolerance to Sulfur Poisoning in CO_2_ Methanation by the Presence of CeO_2_ ,” Applied Catalysis B: Environment and Energy 263 (2020): 118346.

[30] Y. Nishihata , J. Mizuki , T. Akao , et al., “Self‐Regeneration of a Pd‐Perovskite Catalyst for Automotive Emissions Control,” Nature 418 (2002): 164–167, 10.1038/nature00893. 12110885

[31] D. Neagu , G. Tsekouras , D. Miller , et al., “In Situ Growth of Nanoparticles through Control of Non‐Stoichiometr,” Nature Chemistry 5 (2013): 916–923. 10.1038/nchem.1773 24153368

[32] P. Gao , S. Tang , X. Han , et al., “Boosting CO_2_ Methanation via Tuning Metal‐support Interaction over Hollow Ni/CeO_2_ ,” Chemical Engineering Journal 498 (2024): 155784, 10.1016/j.cej.2024.155784.

[33] K. Hong , M. Choi , Y. Bae , et al., “Direct Methane Protonic Ceramic Fuel Cells With Self‐Assembled Ni‐Rh Bimetallic Catalyst,” Nature Communications 14 (2023): 7485, 10.1038/s41467-023-43388-8. PMC10657466 37980343

[34] T. G. Novak , A. E. Herzog , M. R. Buck , et al., “Atomically Dispersed Nickel in CeO_2_ Aerogel Catalysts Completely Suppresses Methanation in the Water‐Gas Shift Reaction,” Science Advances 10 (2024): 9120, 10.1126/sciadv.adr9120. PMC11578172 39565851

[35] F. Liu , H. Deng , Z. Wang , et al., “Synergistic Effects of In‐Situ Exsolved Ni–Ru Bimetallic Catalyst on High‐Performance and Durable Direct‐Methane Solid Oxide Fuel Cells,” Journal of the American Chemical Society 146 (2024): 4704–4715, 10.1021/jacs.3c12121. 38277126

[36] V. P. Pakharukova , D. I. Potemkin , O. A. Stonkus , N. A. Kharchenko , A. A. Saraev , and A. M. Gorlova , “Investigation of the Structure and Interface Features of Ni/Ce_1–x_ Zr_X_ O_2_ Catalysts for CO and CO_2_ Methanation,” The Journal of Physical Chemistry C 125 (2021): 20538–20550, 10.1021/acs.jpcc.1c05529.

[37] R.‐P. Ye , Q. Li , W. Gong , et al., “High‐Performance of Nanostructured Ni/CeO_2_ Catalyst on CO_2_ Methanation,” Applied Catalysis B: Environment and Energy 268 (2019): 118474, 10.1016/j.apcatb.2019.118474.

[38] X. Jiang , X. Li , J. Wang , D. Long , L. Ling , and W. Qiao , “Three‐Dimensional Mn–Cu–Ce Ternary Mixed Oxide Networks Prepared by Polymer‐assisted Deposition for HCHO Catalytic Oxidation,” Catalysis Science & Technology 10 (2018): 2740–2749, 10.1039/C8CY00212F.

[39] M. Li and A. Veen , “Tuning the Catalytic Performance of Ni‐Catalysed Dry Reforming of Methane and Carbon Deposition via Ni‐CeO_2_‐ Interaction,” Applied Catalysis B: Environmental 237 (2018): 641–648, 10.1016/j.apcatb.2018.06.032.

[40] H. Li , Y. Cui , Q. Liu , and W.‐L. Dai , “Insight into the Synergism between Copper Species and Surface Defects Influenced by Copper Content over Copper/Ceria Catalysts for the Hydrogenation of Carbonate,” Chemcatchem 10 (2018): 619–624, 10.1002/cctc.201701384.

[41] S. Sultan , J. N. Tiwari , A. N. Singh , et al., “Single Atoms and Clusters Based Nanomaterials for Hydrogen Evolution, Oxygen Evolution Reactions, and Full Water Splitting,” Advanced Energy Materials 9 (2019): 1900624, 10.1002/aenm.201900624.

[42] S. Anh and A. Manthiram , “Single Ni Atoms and Clusters Embedded in N‐Doped Carbon ‘Tubes on Fibers’ Matrix With Bifunctional Activity for Water Splitting at High Current Densities,” Small 16 (2020): 2002511. 10.1002/smll.202002511 33439543

[43] H. Yan , N. Zhang , and D. Wang , “Highly Efficient CeO_2_‐Supported Noble‐Metal Catalysts: from Single Atoms to Nanoclusters,” Chem Catalysis 2 (2022): 1594–1623, 10.1016/j.checat.2022.05.001.

[44] J. Lee , P. Tieu , J. Finzel , et al., “How Pt Influences H_2_ Reactions on High Surface‐Area Pt/CeO_2_ Powder Catalyst Surfaces,” Journal of the American Chemical Society 3 (2023): 2299–2313. 10.1021/jacsau.3c00330 PMC10466333 37654595

[45] F. Hu , K. Chen , Y. Ling , et al., “Smart Dual‐Exsolved Self‐Assembled Anode Enables Efficient and Robust Methane‐Fueled Solid Oxide Fuel Cells,” Advanced Science 11 (2024): 2306845, 10.1002/advs.202306845. PMC10787062 37985567

[46] Z. Bin , “A State‐Resonant Energy Transmission Law for Energy Materials and beyond,” Energy Z 2 (2026): 200001.

[47] A. Babaei , S. Jiang , and J. Li , “Electrocatalytic Promotion of Palladium Nanoparticles on Hydrogen Oxidation on Ni/GDC Anodes of SOFCs via Spillover,” Journal of The Electrochemical Society 156 (2009): B1022, 10.1149/1.3156637.

[48] S. Lin , L. Kang , L. Gong , et al., “Facet Specificity of Yttrium Doping on CO_2_ Methanation over Ni/CeO_2_ Catalysts,” Chemical Engineering Journal 502 (2024): 157840, 10.1016/j.cej.2024.157840.

