# Supplementary material for: Synergistic Ni Single Atoms/Nanoparticles on CeO2 for High‐Performance and Durable SOFC Hydrogen Electrodes
Source: Adv Sci (Weinh). 2026 Jun 30;13(52):e76282. doi: 10.1002/advs.76282 (PMC13337026; doi:10.1002/advs.76282)
Supplement: Supplementary file 1 [file ADVS-13-e76282-s001.docx]

Supporting Information

**Synergistic Ni Single Atoms/Nanoparticles on CeO_2_ for High-Performance and Durable SOFC Hydrogen Electrodes**

Mengting Liu^+^, Fengyang Yu^+^*, Qian Yang, Ling Huang^1,2,^*

^1^State Key Laboratory of Chemistry and Utilization of Carbon Based Energy Resources;

College of Chemistry, Xinjiang University, Urumqi, 830046, Xinjiang, PR China.

^2^School of Materials Science and Engineering, Nanjing Tech University, Nanjing 211816, China.

E-mail: [iamlhuang@njtech.edu.cn;](mailto:iamlhuang@njtech.edu.cn;) [yufengyang@xju.edu.cn](mailto:yufengyang@xju.edu.cn)

Table of Contents:

1. Experimental Section
2. Supplementary Figures &Tables
3. Reference
1. **Experimental Section**

**Materials**

C_10_H_14_O_4_Ni·2H_2_O (AR, 99%, Aladdin Biochemical Technology), Ce(NH_4_)_2_(NO_3_)_6_ (AR, 99%, Aladdin Biochemical Technology), N,N-dimethylformamide(DMF, AR, 99.5%, Tianjin Zhiyuan), Ethylenediaminetetraacetic acid (H_2_BDC, AR, 99.5%, Aladdin Biochemical Technology), CH_3_OH (AR, 99.5%, Tianjing Zhiyuan), Ni(NO_3_)_2_·6H_2_O (AR, 98%, Xilong Scientific) were used as the anode material. (NH_4_)_2_CO_3_ (40.0%, Tianjing Xingbote), Ce(NO_3_)_3_·6H_2_O (99.95%, Aladdin Biochemical Technology), La(NO_3_)_3_·6H_2_O (99.99%, Aladdin Biochemical Technology) were used as the barrier layer (LDC). LSGM plate (LSGM-8282, 300 μm thickness, WOW Materials) was used as the electrolyte materials. LSCF slurry (La_0.65_Sr_0.4_Co_0.2_Fe_0.8_O_3+δ_, 99.5%, WOW Materials) was used as the cathode material. Terpineol (95%, Aladdin Biochemical Technology), Ethyl cellulose (45-55mPa·s, Aladdin Biochemical Technology), Ceramic sealant (Ceramabond 552, AREMCO), and Silver conducting resin (DAD-87, Shanghai Institute of Synthetic Resin) were used in the fabrication of the cells.

**Characterization**

The material’s morphology, crystal structure, and chemical valence state were identified using X-ray (XRD, Bruker D8 Advance), Thermogravimetric analysis (TG, STA 3700), field emission scanning electron microscope (FESEM, Hitachi S-4800H), high resolution transmission electron microscope (HR-TEM, JEM-2100F), thermo scientific iliad transmission electron microscope(STEM, Thermo Fisher Scientific Themis Z), X-ray photoelectron spectrometer (XPS, Thermo Fisher Scientific K-Alpha), Fe K-edge extended X-ray absorption fine structure (EXAFS, BL 14W Beam line at the Shanghai Synchrotron Radiation Facility ) techniques, Brauner-Emmet-Teller (BET, ASAP 2460), Hydrogen temperature-programmed desorption (H_2_-TPD, AutoChem II 2920), Hydrogen temperature-programmed reduction (H_2_-TPR, AutoChem II 2920).

**Materials Preparation**

Preparation of UiO-66(Ce) ^[1]^. Typically, 35.4 mg of EDTA was dissolved in 1.2 mL of DMF under continuous heating and stirring until complete dissolution. Subsequently, 400 μL of a Ce(NH_4_)_2_(NO_3_)_6_ solution (0.553 M) was added to the mixture. The resulting mixture was then transferred into a Teflon-lined stainless-steel autoclave and heated at 100 °C for 24 h. After cooling to room temperature, the light-yellow precipitate was collected by centrifugation, washed sequentially with DMF and methanol, and finally dried under vacuum at 70 °C for 48 h to obtain a pale-yellow powder.

Preparation of e-Ni/CeO_2_-5%*.* TG analysis on Ce-MOF synthesized by a typical synthetic procedure showed that the weight percentage of pure CeO₂ after calcination at 800 ℃ was 41.803%. C_10_H_14_O_4_Ni·2H_2_O was combined with Ce-MOF in varying molar ratios. Subsequently, 250 mL of CH_3_OH was added to the mixture in a beaker, and the solution was sonicated for 30 minutes. The beaker containing the mixture was then placed in a thermostatic magnetic stirring bath and stirred at 60 ℃ until the entire contents solidified. After solidification, the sample was transferred to a vacuum oven and dried at 60 ℃ for 24 h. The solidified sample was ground in a mortar for 30 minutes until it became a fine powder. This powder was then placed into a muffle furnace and heated gradually at a rate of 4 ℃/min until it reached 800 ℃. The furnace maintained this temperature for 4 h, resulting in the production of CeO_2_ anode powder doped with varying proportions of Ni. Subsequently, the samples were reduced in H_2_ at 800 ℃ for 4 h with a heating rate of 4 ℃/min.

Preparation of i-Ni/CeO_2_-5%. Carefully blend Ni(NO_3_)_2_·6H_2_O with glycine in a precisely determined ratio to ensure the concentration of Ni^2+^ ions in the resultant solution reaches 0.1 mol/L. Use a pipette to uniformly dispense the Ni solution dropwise onto the Ce-MOF, maintaining a consistent immersion volume of 0.5 mL throughout. Then, gently transfer the sample treated with the dropwise Ni solution into a conical flask, subject it to vacuum treatment to remove trapped gases, and dry it in a vacuum drying oven at 90 ℃ to obtain a stable, solidified sample. Finally, the dried sample is treated using the same procedure as e-Ni/CeO_2_-5%.

Preparation of LDC. Measure out a specific mass ratio of Ce(NO_3_)_3_·6H_2_O, La(NO_3_)_3_·6H_2_O and citric acid (The molar ratio of Ce: La is 4:6, and the molar ratio of metal ions to citric acid is 1:1.5). Add an appropriate volume of deionized water to the beaker, then heat and stir the mixture at 80 °C until a gel-like substance forms. Transfer the gel to a muffle furnace and heat it further until it turns into a powder. Place this powder into a muffle furnace and calcine it at 700 °C for 2 h. Finally, grind the calcined powder sample to a fine powder.

**Cell Fabrication**

A single cell featuring an electrolyte support is constructed using LSGM as the electrolyte material, with a thickness of 300 μm. The cathode is composed of LSCF, while LDC serves as the barrier layer. The anode is constructed using various compositions including CeO_2_, e-Ni/CeO_2_-5%, i-Ni/CeO_2_-5%. The specific procedure entails screen printing an LDC barrier layer onto the anode side in order to prevent any unwanted reactions between the electrolyte and the anode. Subsequently, a blend of turpentine terpineol and ethyl cellulose was formulated to create an electrode adhesive, which was then introduced into the anode powder mixture. After meticulous grinding, an anode slurry was formulated and subsequently coated onto one surface of the LDC barrier layer. Utilizing a similar approach, a cathode slurry was prepared and then applied to the opposite side of the LSGM electrolyte, ensuring a working area of approximately 0.28 cm^2^ for the cathode. Following the drying process, the electrolyte sheet was subjected to a gradual heating process in a muffle furnace, reaching a temperature of 1100 ℃ at a rate of 2 ℃/min. This calcination step was maintained for 2 h, resulting in the formation of Anode|LDC|LSGM|LSCF single-cell sheets. Subsequent to this process, the anode side assumes the role of a current collector, with silver wires of 0.2 mm diameter being attached to both ends of the anode, serving as electrical connections. The completed sheet cell is attached to an aluminum oxide tube, positioning the anode side towards the interior. The current collector is then secured using DAD-87 conductive adhesive, and the assembly is sealed with ceramic adhesive to form a single cell.

**Electrochemical Measurement**

Both Electrochemical Impedance Spectroscopy (EIS) and single-cell testing were conducted using the Gamry testing system. For EIS, symmetrical cells were tested in wet H_2_ (3 vol% H_2_O). Prior to testing, the symmetrical cells were pre-reduced in a H_2_ atmosphere at 800 ℃ for 3 h. Subsequently, EIS was performed on the cells within a temperature range of 700-800 ℃, with the frequency range being set from 10^6^ Hz to 0.01 Hz. For single-cell testing, the anode was pre-reduced in a H_2_ atmosphere at 800 ℃ for 3 h. The cell was then tested in wet H_2_ (3 vol% H_2_O), with the cathode side being exposed to air. During testing, the temperature was maintained between 700-800 ℃, and the frequency range was set from 10^6^ Hz to 0.01 Hz.

2. Supplementary Figures &Tables

**FIGURE S1.** XRD patterns of UiO-66(Ce) powders.

**FIGURE S2.** The elemental mapping results of (a-d) e-NiO/CeO_2_-5% powder, (e-h) e-Ni/CeO_2_-5% powder, and (i-l) i-Ni/CeO_2_-5% powder.

**FIGURE S3.** XRD patterns of (a) Ni/CeO_2_ and (b) NiO/CeO_2_ powders.

**FIGURE S4.** k-space of the e-Ni/CeO_2_-5%, NiO, Ni foil.

**FIGURE S5.** WT-EXAFS of the (a) e-Ni/CeO_2_-5%, (b) Ni foil, (c) NiO.

**FIGURE S6.** XPS survey spectrum of CeO_2_, e-Ni/CeO_2_-5% and i-Ni/CeO_2_-5%.

**FIGURE S7.** Cross sectional SEM images of electrolyte-supported symmetrical cell after EIS testing, where LDC, LSGM and Ni/CeO_2_ work as the barrier layer, electrolyte, and anode, respectively.

**FIGURE S8.** The EIS of symmetric cells with Ni/CeO_2_ at (a) 800, (b) 750, (c) 700 °C in wet H_2_ (3 vol% H_2_O). (d) The R_p_ of e-Ni/CeO_2_-5% and i-Ni/CeO_2_-5%.

**FIGURE S9.** Cross sectional SEM images of electrolyte-supported single cell after *I-V* testing, where LSCF, LDC, LSGM and Ni/CeO_2_ work as the cathode, barrier layer, electrolyte, and anode, respectively.

**FIGURE S10.** *I-V* and *I-P* curves of single cells with the Ni/CeO_2_ anodes in wet H_2_ (3 vol% H_2_O) at (a) 800, (b) 750 and (c) 700 °C. EIS spectra of the single cell with Ni/CeO_2_ anode operated in wet H_2_ (3 vol% H_2_O) at (d) 800, (e) 750 and (f) 700 °C.

**FIGURE S11.** (a) Nyquist plot and equivalent circuit fitting for e-Ni/CeO_2_-5%. (b) The DRT of e-Ni/CeO_2_-5% measured at 800 °C in varying PH_2_.

**FIGURE S12.** Reaction mechanism of the hydrocarbon fuel oxidation on the surface of e-Ni/CeO_2_-5% with fine Ni SAs/NPs.

**FIGURE S13.** (a-d) SEM images and elemental maps of the anode regions of the cells with i-Ni/CeO_2_-5% before and after the stability tests. (e-h) SEM images and elemental maps of the anode regions of the cells with e-Ni/CeO_2_-5% before and after the stability tests.

Table S1. Polarization resistances (R_P_) of symmetrical cells with the Ni/CeO_2_ anodes in wet hydrogen (3% H_2_O) at 700-800°C

| Rp (Ω cm^2^)  Anodes | 800 ℃ | 750 ℃ | 700 ℃ |
| --- | --- | --- | --- |
| i-Ni/CeO_2_-5% | 0.149 | 0.257 | 0.494 |
| e-Ni/CeO_2_-5% | 0.098 | 0.147 | 0.259 |

Table S2. PPDs of single cells with the Ni/CeO_2_ anodes in humidified hydrogen (3% H_2_O) at 700-800°C

| PPD (W cm^-2^)  Anodes | 800 ℃ | 750 ℃ | 700 ℃ |
| --- | --- | --- | --- |
| i-Ni/CeO_2_-5% | 0.90 | 0.66 | 0.39 |
| e-Ni/CeO_2_-5% | 1.46 | 0.93 | 0.56 |

Table S3. EIS of single cells with the Ni/CeO_2_ anodes in wet H_2_ (3% H_2_O) at 700-800°C

| EIS (Ω cm^2^)  Anodes | 800 ℃ | | 750 ℃ | | 700 ℃ | |
| --- | --- | --- | --- | --- | --- | --- |
|  | R_o_ | R_p_ | R_o_ | R_p_ | R_o_ | R_p_ |
| i-Ni/CeO_2_-5% | 0.177 | 0.158 | 0.212 | 0.235 | 0.310 | 0.392 |
| e-Ni/CeO_2_-5% | 0.113 | 0.099 | 0.162 | 0.153 | 0.245 | 0.266 |

Table S4. Summary of PPDs for different SOFC anode materials of the electrolyte-supported cells in wet H_2_ (3% H_2_O).

| Anode | Electrolyte | Cathode | PPD  (W cm^-2^) | Ref. |
| --- | --- | --- | --- | --- |
| La_2_NiO_4_-infiltrated SDC | SDC  (300 μm) | SDC | 0.55 | [2] |
| GDC+TiO_0.2_C_0.8_ | GDC  (510 μm) | GDC+LSCF  (La_0.6_Sr_0.2_Co_0.8_Fe_0.2_O_3−δ_) | 0.13 | **[3]** |
| Sr_1.8_La_0.2_FeMoO_6-δ_ | GDC  (300 μm) | LSCF | 0.885 | [4] |
| NiO-GDC | GDC  (220 μm) | LSCF-GDC | 0.37 | [5] |
| Ni-30 wt%GDC | SDC  (150 μm) | LSCF | 0.73 | [6] |
| Sr_2_Fe_1.5_Mo_0.5_O_6-δ_ | LSGM  (265 μm) | Sr_2_Fe_1.5_Mo_0.5_O_6-δ_ | 0.835 | [7] |
| Pr_0.4_Sr_0.6_Co_0.2_Fe_0.7_Nb_0.1_O_3-δ_ | LSGM  (300 μm) | Pr_0.8_Sr_1.2_(Co,Fe)_0.8_Nb_0.2_O_4+δ_ -Co/Fe | 0.78 | [8] |
| PrBaMn_2_O_5+δ_ | LSGM  (300 μm) | NBSCF-GDC | 1.57 | [9] |
| Fe/LSMF-GDC | LSGM  (280 μm) | LSCF-GDC | 0.75 | [10] |
| FeRu/STFRu | LSGM  (300 μm) | LSCF-GDC | 0.7 | [11] |
| Ni-SrTi_0.3_Fe_0.7_O_3-δ_ | LSGM  (300 μm) | LSCF-GDC | 1.2 | [12] |
| Ni/SGNM28-GDC | LSGM  (280 μm) | LSCF-GDC | 0.67 | [13] |
| R-Pr_0.5_Ba_0.2_Sr_0.3_FeO_3-δ_ | LSGM  (250 μm) | A-Pr_0.5_Ba_0.2_Sr_0.3_FeO_3−δ_ | 1.23 | [14] |
| Ni-Sr_3_Fe_1.5_Mo_0.5_O_7-δ_ | LSGM  (400 μm) | LSCF-GDC | 0.49 | [15] |
| NiFe-Pr_0.8_Sr_1.2_Ni_0.2_Fe_1.3_Mo_0.5_O_6-δ_-GDC | LSGM  (200 μm) | PrBa_0.5_Sr_0.5_Co_1.5_Fe_0.5_O_6-δ_ | 1.45 | [16] |
| Ru-SrMo_0.9_O_3-δ_ | LSGM  (300 μm) | LSCF | 0.84 | [17] |
| Sr_2_Fe_1.2_Co_0.3_Mo_0.5_O_6-δ_ | LSGM  (200 μm) | Sr_2_Fe_1.2_Co_0.3_Mo_0.5_O_6-δ_ | 1.0 | [18] |
| **e-Ni/CeO_2_-5%** | **LSGM**  **(300 μm)** | **LSCF** | **1.46** | **This work** |

3. Reference
1. M. Lammert, M. Wharmby, S. Smolders, et al., “Cerium-Based Metal Organic Frameworks with UiO-66 Architecture: Synthesis, Properties and Redox Catalytic Activity,” *Chemical Communications 51* (2015): 12578.
2. G. Yang, C. Su, R. Ran, et al., “Advanced Symmetric Solid Oxide Fuel Cell with an Infiltrated K_2_NiF_4_-Type La_2_NiO_4_ Electrode,” *Energy Fuels* 28 (2014): 356.
3. A. Sinha, D. Millera, J. Irvine, “Development of Novel Anode Material for Intermediate Temperature SOFC (IT-SOFC),” *Journal of Materials Chemistry A* 4 (2016): 11117-11123.
4. X. Yang, J. Chen, D. Panthi, et al., “Electron Doping of Sr_2_FeMoO_6-δ_ as High Performance Anode Materials for Solid Oxide Fuel Cells,” *Journal of Materials Chemistry A 7* (2019): 733-743.
5. F. Wang, Y. Liu, J. Sun, et al., “Boosting Electrochemical Performance of a Nanocomposite Ni-GDC Anode via Oxygen Vacancy and Ni Dispersion Modulation for Solid Oxide Fuel CellsClick to copy article link,” *ACS Applied Energy Materials* 6 (2023): 9409-9416.
6. B. Wang, Z. Yue, Z. Chen, et al., “Facile Construction of Nanostructured Cermet Anodes with Strong Metal–Oxide Interaction for Efficient and Durable Solid Oxide Fuel Cells,” *Small* 19 (2023): 2304425.
7. Q. Liu, X. Dong, G. Xiao, et al., “A Novel Electrode Material for Symmetrical SOFCs,” *Advanced Materials* 22 (2010): 5478-5482.
8. C. Yang, Z. Yang, C. Jin, et al., “Sulfur-Tolerant Redox-Reversible Anode Material for Direct Hydrocarbon Solid Oxide Fuel Cells,” *Advanced Materials* 24 (2012): 1439-1443.
9. S. Sengodan, S. Choi, A. Jun, et al., “Layered Oxygen-Deficient Double Perovskite as an Efficient and Stable Anode for Direct Hydrocarbon Solid Oxide Fuel Cells,” *Nature Materials* 14 (2015): 205-209.
10. Y. Chung, T. Kim, T. Shin, et al., “In Situ Preparation of a La_1.2_Sr_0.8_Mn_0.4_Fe_0.6_O_4_ Ruddlesden–Popper Phase with Exsolved Fe Nanoparticles as an Anode for SOFCs,” *Journal of Materials Chemistry A* 5 (2017**)**: 6437-6446.
11. R. Glaser, T. Zhu, H. Troiani, et al., “The Enhanced Electrochemical Response of Sr(Ti_0.3_Fe_0.7_Ru_0.07_)O_3−_δ Anodes due to Exsolved Ru–Fe Nanoparticles,” *Journal of Materials Chemistry A* 6 (2018): 5193-5201.
12. T. Zhu, H. Troiani, L. Mogni, et al., “Ni-Substituted Sr(Ti,Fe)O_3_ SOFC Anodes: Achieving High Performance via Metal Alloy Nanoparticle Exsolution,” *Joule* 2 (2018): 478-496.
13. K. Kim, M. Rath, H. Kwak, et al., “A Highly Active and Redox-Stable SrGdNi_0.2_Mn_0.8_O4_±δ_ Anode with in Situ Exsolution of Nanocatalysts,” *ACS Catalysis* 9 (2019): 1172-1182.
14. H. Kim, C. Lim, O. Kwon, et al., “Unveiling the Key Factor for the Phase Reconstruction and Exsolved Metallic Particle Distribution in Perovskites,” *Nature Communications* 12 (2021): 6814.
15. X. Zhang, Y. Tong, T. Liu, et al., “Robust Ruddlesden-Popper Phase Sr_3_Fe_1.3_Mo_0.5_Ni_0.2_O_7-δ_ Decorated with In-Situ Exsolved Ni Nanoparticles as an Efficient Anode for Hydrocarbon Fueled Solid Oxide Fuel Cells,” *SusMat* 2 (2022): 487-501.
16. T. Tan, Z. Wang, K. Huang, et al., “High-Performance Co-production of Electricity and Light Olefins Enabled by Exsolved NiFe Alloy Nanoparticles from a Double-Perovskite Oxide Anode in Solid Oxide-Ion-Conducting,” Fuel Cells, *ACS Nano* 17 (2023): 13985-13996.
17. V. Cascos, M. Lacaba, N. Biskup, et al., “SrMo_0.9_O_3−δ_ Perovskite with Segregated Ru Nanoparticles Performing as Anode in Solid Oxide Fuel Cells,” *ACS Applied Materials & Interfaces* 16 (2024): 17474-17482.
18. H. Jeon, Y. Kim, H. Kim, et al., “Optimizing Reversible Exsolution and Phase Transformation in Double Perovskite Sr_2_Fe_1.5-x_Co_x_Mo_0.5_O_6-δ_ Electrodes for High-Performance Symmetric Solid Oxide Cells,” *Small* 20 (2024): 2401628.
